# Mental Pain in Eating Disorders: An Exploratory Controlled Study

**DOI:** 10.3390/jcm10163584

**Published:** 2021-08-14

**Authors:** Elena Tomba, Lucia Tecuta, Valentina Gardini, Elena Lo Dato

**Affiliations:** Department of Psychology, University of Bologna, 40127 Bologna, Italy; lucia.tecuta2@unibo.it (L.T.); valentina.gardini8@unibo.it (V.G.); elena.lodato@studio.unibo.it (E.L.D.)

**Keywords:** mental pain, eating disorders, suicidality, assessment, depression

## Abstract

Mental pain (MP) is a transdiagnostic feature characterized by depression, suicidal ideation, emotion dysregulation, and associated with worse levels of distress. The study explores the presence and the discriminating role of MP in EDs in detecting patients with higher depressive and ED-related symptoms. Seventy-one ED patients and 90 matched controls completed a Clinical Assessment Scale for MP (CASMP) and the Mental Pain Questionnaire (MPQ). ED patients also completed the Beck Depression Inventory-II (BDI-II), Clinical Interview for Depression (CID-20), and Eating Attitudes Test (EAT-40). ED patients exhibited significantly greater severity and higher number of cases of MP than controls. Moreover, MP resulted the most important cluster predictor followed by BDI-II, CID-20, and EAT-40 in discriminating between patients with different ED and depression severity in a two-step cluster analysis encompassing 87.3% (*n* = 62) of the total ED sample. Significant positive associations have been found between MP and bulimic symptoms, cognitive and somatic-affective depressive symptoms, suicidal tendencies, and anxiety-related symptoms. In particular, those presenting MP reported significantly higher levels of depressive and anxiety-related symptoms than those without. MP represents a clinical aspect that can help to detect more severe cases of EDs and to better understand the complex interplay between ED and mood symptomatology.

## 1. Introduction

Mental pain (MP) is progressively gaining clinical relevance in the field of psychiatry and clinical psychology [1,2,3,4]. It could be described as a subjective, overwhelming, and unbearable experience characterized by a number of uncomfortable emotions—such as anguish, anger, emptiness, agitation, and guilt—which arises from negative self-evaluations and self-awareness of one’s own inabilities and failures [1,2,3,4]. Over the past decades, it has also been present in the literature as suffering [5,6], psychic pain, psych-ache, emotional pain, psychological pain, social pain, emptiness, or internal perturbation [1,7,8,9]. There is growing evidence supporting the idea that MP may represent an independent condition with its own neurobiological characteristics [10]. However, it is significantly associated with several psychopathological conditions, such as suicidal ideation [2,7,11], depression [10,12,13], anxiety [14,15], borderline personality disorder [16], and emotion dysregulation [9].

More recently, based on the available literature stemming from the psychosomatic framework and clinical setting, Fava and colleagues [17] proposed an operationalization of the definition of MP as a transdiagnostic construct, supported by its association with several psychiatric illnesses, characterized by specific clinical features, such as feeling pain, feeling of being wounded, sense of hopelessness and helplessness, lack of localization in the body, persistence in time, lack of understanding of its occurrence, feelings of emptiness, loss of meaning, irreversibility of the pain, and suicidality. A clinimetric assessment tool was also empirically developed [18,19] and validated [20] and classified as a Patient-Reported Outcome (PRO), that is, any self-rated and “easy to use” report coming directly from patients about how they perceive symptoms and how they function or feel in relation to a health condition [21,22,23,24]. Extending the assessment to PRO is increasingly necessary in psychiatry to identify at-risk patients as well as to detect the subjective impact and burden of symptoms on patients [25]. According to Fava and colleagues [17], MP should be incorporated into current psychiatric nosography as a specifier of the DSM-5 “clinically significant distress” caused by symptoms of a psychiatric disorder [26]. Indeed, MP has been found to be associated with worst levels of psychopathology and to distinguish between patients of diverse clinical populations [20]. In particular, higher levels of MP have been found in migraine outpatients with comorbid depression, suicidal tendencies, hopelessness, and guilt feelings [20]. In primary care, MP more frequently characterize patients with at least one psychiatric diagnosis based on the DSM-5 (in particular mood disorders) or with at least one syndrome based on the Diagnostic Criteria for Psychosomatic Research (DPCR) (in particular demoralization or irritable mood) [14].

Despite the clinical relevance of MP, there are some psychiatric populations in which it has not yet been investigated. Specifically in eating disorders (EDs), it is unexplored, although its inclusion may yield important clinical contributions to the standard assessment in this population. EDs are indeed not only characterized by a disturbance of eating and eating-related behaviors but are often comorbid with difficulties in tolerating negative emotional states [27,28], emotion dysregulation [29], and other psychiatric comorbidities, the most common being major depression [30,31], with suicide being one of the most frequently reported causes of death [32]. As these clinical features also characterize MP [2,7,9,10,11,33,34], adding the evaluation of MP in the assessment of EDs may support clinicians in better understanding the complex interplay between specific ED and non-specific ED symptomatology, in particular mood symptomatology. This is important considering that detecting ED patients at higher risk for depression and suicidality is increasingly urgent given the recent prevalence estimates of suicide attempts in U.S. adults with lifetime DSM-5 EDs, which range from 15.7% for anorexia nervosa restricting subtype (AN-R) and 22.9% in binge-eating disorder (BED) to 44.1% for anorexia binging-purging subtype (AN-BP) [35]. Moreover, since MP is considered a PRO, its measurement can offer a more in-depth look into the subjective experience of patients related to the burden of the ED illness [21,22,23,24,25].

Thus, to broaden the evaluation of psychological distress in EDs, the aims of the current study are to explore the presence of MP in patients with EDs when compared to controls and to examine the clinical utility of measuring MP in discriminating between ED patients in terms of eating and depressive symptomatology, including suicidality. We also aim to evaluate the presence of associations between MP, depressive symptomatology, and eating symptomatology and to compare ED patients with comorbid MP with those without MP in these variables.

## 2. Materials and Methods

### 2.1. Participants

Participants were enrolled in the frame of a larger study aimed at assessing psychological and psychosomatic features in ED patients [36]. Consecutively screened patients (*n* = 74), both inpatients and outpatients, who met diagnostic criteria for EDs (DSM-5; American Psychiatric Association, 2013), such as anorexia nervosa (AN), bulimia nervosa (BN), binge-eating disorder (BED), and other specified feeding or eating disorder (OSFED), were contacted from a specialized ED treatment center in Bologna, Northern Italy. With the exception of three patients who refused to participate, all invited patients took part in the study (*n* = 71) and were assessed before commencing treatment. Inclusion criteria were: (a) 18–65 years of age (b) with a diagnosis of AN, BN, BED, or OSFED (c) within one month of beginning treatment. The exclusion criteria were: (a) lack of capacity to consent for research, (b) ED diagnosis secondary to a physical health or metabolic condition, (c) comorbid drug/alcohol abuse, psychotic or neurocognitive disorders, and pregnancy. The socio-demographic and clinical data of the ED sample appear in Table 1.

Control participants matched for gender and age were recruited online from the adult general population and from university campuses in Northern Italy with the following inclusion criteria: (a) 18–65 years of age and (b) no prior diagnosis of any ED according to DSM-5 diagnostic criteria. Exclusion criteria were (a) lack of capacity to consent for research and (b) lifetime history of EDs according to DSM-5 diagnostic criteria either as primary diagnosis or in comorbidity to other mental health or due to a physical condition. The project was approved by University of Bologna Bioethics Committee and Department of Psychology Ethics Committee. Informed consent was obtained from all participants included in the study.

### 2.2. Procedures

The evaluation of ED patients was performed during the first intake visit before commencing treatment. ED diagnoses were established at intake by the consensus of a psychiatrist and a clinical psychologist independently using the Structured Clinical Interview for DSM-5 (SCID-5) [37]. Depressive disorder diagnoses were also established with DSM-5 criteria (SCID-5) [37] by independent raters. Each diagnostic interview was conducted and recorded by a clinical psychologist expert in assessment (E.T.) and subsequently reviewed by a consulting psychiatrist specialized in EDs who confirmed the diagnosis. Consent to be recorded while interviewed was obtained from all participants.

### 2.3. Measures

Both ED patients and controls were assessed before commencing treatment through the following instruments:

1. Mental Pain Questionnaire (MPQ) [18,19] to evaluate self-reported MP severity: the MPQ is a 10-item yes/no self-report questionnaire with a total score ranging from 0 to 10 developed to assess the experience of MP based on clinimetric properties. Ten aspects of MP in clinical settings have been identified on the basis of the literature and have been transformed into ten items that constitute the scale: (1) presence of mental pain; (2) feeling of woundedness, an aspect that is clearly defined by the expression “my heart is broken”; (3) the belief that it is not possible to receive any support or help by others (helplessness) and that the situation will not change in the future (hopelessness); (4) pain localization, which cannot be located in a specific part of the body and that is also defined as “central pain”; (5) pain duration; (6) association with specific events or situations that can be identified by the person as the exact moment in which suffering began; (7) feelings of emptiness; (8) loss of life meaning; (9) irreversibility of pain, often accompanied by fears and suffering intolerance; and (10) association with suicidal ideation [17]. The Italian validation study of the MPQ showed good clinimetric properties with excellent reliability for mental pain intensity, especially for moderate/high levels of intensity, with a Cronbach’s alpha of 0.77 [20].
2. Clinical Assessment Scale for Mental Pain (CASMP) (copyright Fava, Tossani, 2012 in Tossani, 2013) [9] to evaluate the presence of observer-assessed MP: this observer-assessed scale is used to assess MP experienced by patients based on their verbal expressions, which can give some information about the description, intensity, temporal patterns associated with physiological and behavioral processes related to the pain. Individuals are asked by a trained clinical psychologist to describe their MP and suffering and its duration (e.g., if it is experienced in specific moments, every day, or less frequently), to compare it to physical pain, if there is anything that ameliorates or that worsens the feeling, and if it is associated with a desire to die or if death is perceived as the only solution to stop MP. The structure of the questions and the ratings of such clinical assessment for MP are based upon the Clinical Interview for Depression (CID-20) [38,39] (see below in this section for more details), with a score ranging from 1 to 7 as follows: absent, very mild or occasional, mild (it comes at moments and then goes away), moderate (it tends to be steady), marked (it hurts all the time and does not get better), severe (it is unbearable), and extreme (it makes you feel like you want to die) [9]. As with the CID-20, a score of 3 or above in the individual items was considered the cut-off for presence of the experienced MP.

Only ED patients were also assessed through the following instruments:

3. Eating Attitudes Test-40 (EAT-40) [40] to assess self-reported ED symptomatology: a 40-items self-report screening measure identifying behaviors and cognitive patterns associated with eating disorders, where a greater total score indicates greater eating disorder severity. The measure yields a total score and three subscales’ scores: dieting, bulimia and food preoccupations, and oral control. The measure shows excellent psychometric properties [40]. In this study, we used the Italian version of the EAT-40, which has been validated [41] and exhibits good psychometric properties, with reported Cronbach alphas of 0.80 for dieting subscale, 0.70 for food and bulimic preoccupations subscale, and 0.83 for oral control subscale.
4. Beck Depression Inventory-II (BDI-II) [42] to measure self-reported depression: a 21-item questionnaire. A total score ranging 0–63 is an index of depression severity, with higher total scores indicating more severe depressive symptoms. Composite scales of cognitive and somatic-affective symptoms were calculated. The measure exhibits excellent psychometric properties across clinical populations [43]. An average Alpha coefficient of 0.9 has been found, indicating a good internal consistency of the scale as well as a good retest reliability, with Pearson’s r coefficients ranging from 0.73 to 0.96. Moreover, the BDI-II has a good convergent and discriminative validity, displaying a good capacity in discriminating between patients with and without depression [43] and between patients and nonclinical individuals [44]. In this study, we used the Italian version of the BDI-II [45].
5. Clinical Interview for Depression-20 item interview (CID-20) [38,39] to evaluate observer-assessed depression symptomatology severity: CID-20 is a dimensional observer-rated assessment instrument that consists of an expanded version of the Hamilton Rating Scale for Depression [46]. The interview covers 20 symptom areas/scales and is conducted by a trained clinical psychologist. Each area is rated on a 1–7 point scale, with 1 indicating absence of symptoms and 7 severe incapacitating manifestations. A score of 3 or above in the individual items was considered the cut-off for presence of the symptom. The scale encompasses a wide range of symptoms (such as irritability and phobic anxiety) compared to other scales and is particularly suitable to assess subclinical symptoms of mood disorders [25,39,47]. Summed total score and scores for anxiety and depression can be calculated, and individual items are also suitable for use as separate measures [39]. The CID-20 showed excellent psychometric and clinimetric properties in terms of inter-rater reliability (Cohen k ranging from 0.81 to 0.82), discriminant validity, sensitivity to changes with treatment, test-retest reliability (depression r = 0.58; anxiety r = 0.59), and concurrent and divergent validity [38,39]. The Italian version showed clinimetric and psychometric properties consistent with the English version [48]. For the current study, CID-20 items pertaining to depressive symptoms with the highest frequency (>30%) in EDs were included [36]. Two items concerning appetite and weight gain/loss (items 12 and 13) were omitted due to the potentially confounding aspects of eating disorder-related symptomatology.
6. In ED patients, body mass index (BMI), illness duration in months, and use of antidepressant therapy were furthermore collected from updated medical records.

### 2.4. Data Analyses

Descriptive statistics were run for socio-demographic (age, education, level of occupation, marital status) and clinical characteristics (BMI, illness duration, use of antidepressants, ED diagnoses, depressive disorders diagnoses). Independent *t*-tests and chi-square tests were run to compare ED patients and control participants in MPQ and CASMP.

In the ED group, a two-step cluster analysis was also performed in order to organize the sample into two or more mutually exclusive groups of participants sharing common properties [49] and to see whether MP would be a significant cluster predictor. Variables included in the cluster analysis were MPQ, CASMP, CID-20, EAT-40, and BDI-II total scores. The log-likelihood distance measure was used, and no prescribed number of clusters was suggested a priori. The Bayesian Information Criterion (BIC) was used to judge the final cluster solution.

Correlation analyses using Pearson’s r were run to observe the association between MPQ and CASMP and BDI-II, CID-20, and EAT-40 subscales.

Independent *t*-tests and chi-square tests were run to compare ED patients with comorbid MP with those without in socio-demographic and clinical characteristics. Multivariate Analyses of Variance (MANOVA) with Bonferroni corrections were conducted to compare ED patients with comorbid MP with those without in CID-20, BDI-II, and EAT-40 subscales while controlling for age, BMI, and illness duration.

Prior to performing all statistical analyses, normal distribution of all variables was tested by using the Shapiro–Wilk test. All variables showed a normal distribution, so statistical analyses were performed.

In all analyses, the level of significance was set at *p* < 0.05 (two-sided). The Statistical Package for Social Sciences (SPSS; IBM Corp., Armonk, NY) was used for all calculations.

## 3. Results

### 3.1. ED Patient Sample

The patient response rate was high, with 95.95% (*n* = 71) of ED patients out of 74 agreeing to participate. The 71 ED patients were all females, with a mean age of 28.16 ± 11.29 years and mean educational years of 14.19 ± 3.21. In all, 56.3% (*n* = 40) had a diagnosis of AN, 18.3% (*n* = 13) of BN, 14.1% (*n* = 10) of BED, and 11.3% (*n* = 8) of OSFED. A total of 52.1% (*n* = 37) of the patients were outpatients, and the remaining 47.9% (*n* = 34) were inpatients. ED outpatients and inpatients did not differ significantly in the main socio-demographic characteristics, that is, age, education, or BMI (Table 1). They differed significantly in illness duration (t(67) = −0.2.49, *p* = 0.015), with inpatients reporting longer length of illness (141.03 ± 110.24 months) compared to outpatients (77.14 ± 102.30 months) and lower level of occupation ($\chi^{2}$(14) = 82.48, *p* < 0.01). About a third (*n* = 20, 29.85%) of patients were currently on antidepressants, more specifically on selective serotonin-reuptake inhibitors (SSRI). A total of 55.88% (*n* = 38) of ED patients had a comorbid diagnosis of depression, including major depressive disorder (MDD) and persistent depressive disorder (PDD), whereas 44.12% (*n* = 30) did not; inpatients reported significantly higher cases of comorbid depression, as shown in Table 1.

### 3.2. Control Sample

Ninety participants from the general population matched for age and gender constituted the control sample of the study. The control group was all female, with a mean age of 29.36 ± 12.30 and mean educational years of 15.40 ± 3.368. Significant differences were found between ED patients and controls in the levels of education (t(151) = −2.22, *p* = 0.02) and occupation ($\chi^{2}$(7) = 44.53, *p* < 0.01) (Table 1).

### 3.3. Comparisons between ED Patients and Controls in Mental Pain

ED patients and controls significantly differed in the number of MP cases assessed through the CASMP (cut-off point >3) [20,36,39] ($\chi^{2}$(1) = 33.872, *p* < 0.01). In particular, a considerable portion of ED patients reported MP (*n* = 31, 43.66%), while half of the ED sample did not (*n* = 40, 56.34%). In the control sample, only a small number of participants reported MP (*n* = 5, 5.6%), whereas the majority of them did not (*n* = 85, 94.4% of the sample). ED patients exhibited also significantly higher scores in MP assessed through the MPQ and the CASMP compared to controls (Table 2).

### 3.4. Two-Step Cluster Analysis in the ED Sample

**Figure 1:**
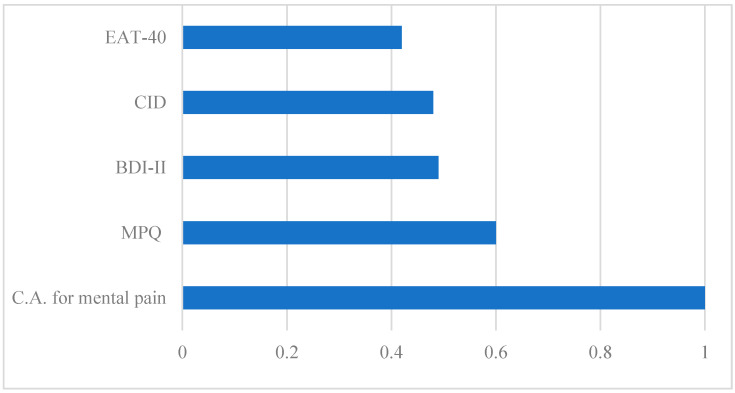
Predictor importance of the variables used in the cluster analysis. Abbreviations: BDI-II, Beck Depression Inventory-II; C.A., Clinical Assessment; CID-20, Clinical Interview for Depression-20 items; EAT-40, Eating Attitudes Test-40; MPQ, Mental Pain Questionnaire.

Two-step cluster analysis performed in the ED sample resulted in two clusters (BIC = 231.858) encompassing 87.3% (*n* = 62) of the sample, with nine outliers. MP assessed with MPQ and with the CASMP emerged as the most important cluster predictors, followed by BDI-II, CID-20, and EAT-40 in discriminating between ED patients (Figure 1). The first cluster had 50% of cases (*n* = 31) and contained ED patients with low MP (MPQ = 2.52 ± 2.00; CASMP = 1.23 ± 0.62), low depression (CDI = 39.19 ± 10.50; BDI-II = 17.71 ± 11.36), and low ED symptomatology (EAT-40 = 41.35 ± 26.30). The second cluster had 50% of cases (*n* = 31) and included ED patients with high MP (MPQ = 5.74 ± 2.34; CASMP = 3.29 ± 1.16), high depression (CID-20 = 53.81 ± 11.64; BDI-II = 31.42 ± 9.21), and high ED symptomatology (EAT-40 = 86.48 ± 46.50), Table 3.

### 3.5. Correlations between MP, ED, and Depressive Symptomatology in ED Patients

Regarding ED symptomatology, only a significant correlation emerged between the CASMP total score and EAT-bulimia and food preoccupations subscale (r = 0.329, *p* = 0.007). Regarding self-rated depressive symptomatology, both somatic-affective (r = 0.340, *p* = 0.005) and cognitive (r = 0.308, *p* = 0.013) subscales of the BDI-II correlated significantly with CASMP scores but not with MPQ. However, as can be observed in Table S1, observer-rated depressive symptoms (CID-20), primarily CID-20-depressed mood (MPQ: r = 0.341, *p* = 0.004; CASMP: r = 0.400, *p* = 0.001), CID-20-suicidal tendencies (MPQ: r = 0.493, *p* < 0.001; CASMP: r = 0.292, *p* = 0.015), CID-20-work and interests (MPQ: r = 0.262, *p* = 0.030; CASMP: r = 0.290, *p* = 0.016), CID-20-general anxiety (MPQ: r = 0.297, *p* = 0.013; CASMP: r = 0.460, *p* < 0.001), CID-20-somatic anxiety (MPQ: r = 0.332, *p* = 0.005; CASMP: r = 0.407, *p* = 0.001), and CID-20-early insomnia (MPQ: r = 0.276, *p* = 0.022; CASMP: r = 0.403, *p* = 0.001), were found to present similar correlations with both CASMP and MPQ (Table S1).

### 3.6. Comparisons between ED Patients with Comorbid MP and without Comorbid MP in Sociodemographic and Clinical Characteristics

ED patients with comorbid MP (*n* = 31) (CASMP score >3) compared to those without MP (*n* = 40) (CASMP score <3) did not differ significantly in the main sociodemographic and clinical characteristics, such as BMI, illness duration, antidepressant use, and being outpatients or inpatients (Table 4). However, among patients considering specific ED diagnoses, AN patients reported significantly more frequent rates of MP (F = 9.457, *p* = 0.02). Moreover, while roughly a third of the clinical sample presented both MP and a concurrent depressive disorder (Major Depression Disorder or Persistent Depressive Disorder), a third was unaffected by either, and a portion of the sample (10.29%, *n* = 7) reported MP in absence of a depressive disorder. Additionally, 22.06% (*n* = 15) did not report MP despite receiving a diagnosis of a depressive disorder, Table 4.

### 3.7. Comparisons between ED Patients with Comorbid MP and without Comorbid MP in Eating and Depressive Symptomatology

Three multivariate analyses of variance (MANOVA) were conducted in order to compare patients with comorbid MP (*n* = 31) and patients without MP (*n* = 40) in ED symptomatology and in depressive symptomatology controlling for age, BMI, and illness duration. Given the high numbers of comparisons, Bonferroni correction was performed, and new *p* values were set (see Table S2).

MANOVA showed no significant differences between the two groups in EAT-40 scales (Table S2). ED patients with MP presented significantly greater BDI-II somatic-affective (F = 9.910, *p* = 0.003) and cognitive depressive (F = 6.210, *p* = 0.016) symptoms and CID-20 depressed mood (F = 10.713, *p* = 0.002), CID-20 general anxiety (F = 17.630, *p* = 0.0001), and CID-20 early insomnia (F = 10.216, *p* = 0.002) than ED patients without comorbid MP (Table S2).

## 4. Discussion

MP has, to the best of our knowledge, never been explored in EDs before. Therefore, the present study was carried out in order to explore the presence of MP in patients with EDs when compared to controls and to examine whether MP can discriminate between ED patients in terms of eating and depressive symptomatology. Associations between MP, depressive symptomatology, and eating symptomatology were also evaluated in the ED sample along with differences between ED patients with comorbid MP and those without MP in these variables. Our findings suggest that MP does not only characterize ED patients when compared to controls but can also represent an important clinical marker for discriminating, within ED patients, subgroups of patients with different levels of depressive and ED-related symptomatology. Significant associations were found especially between MP and disinhibited eating behavioral aspects, such as bulimic symptoms, and between MP and many aspects of depressive-symptomatology, such as suicidal tendencies, general and somatic anxiety, and insomnia. When comparing ED patients with comorbid MP to those without comorbid MP, only higher levels of depressive symptomatology and general anxiety have been found to be different between the groups.

Compared to controls, ED patients reported greater intensity and significantly higher number of cases of MP, confirming previous studies in which higher levels of MP have been established in clinical populations when compared to healthy controls, such as in patients with a major depressive episode [50] or who met the criteria for medically serious suicide attempts [51,52]. The higher cases and levels of MP in EDs compared to controls may be related to the specific difficulties of ED patients in recognizing, regulating, and communicating emotions. As described by patients themselves [53], dysfunctional eating behaviors are often used as a strategy to regulate or avoid uncomfortable emotions and deal with psychological pain. Moreover, alexithymia [54,55], interoceptive awareness deficits [56,57], and emotional dysregulation [58,59], widely known clinical features of EDs, are associated with MP as well [13,33,60].

MP does not only characterize ED patients when compared to controls, but it has been found to be a significant cluster predictor in discriminating two subgroups of ED patients with different levels of severity in depressive and ED symptomatology. Consistently, the group of patients with higher levels of MP also reported higher levels of depressive and ED symptomatology. On the one hand, such findings support the well-known association between MP and depression [10,12,13], depression severity, cognitive depressive symptoms [2,14,50], and suicide among different clinical samples [12,34,50,61,62]. On the other, our results are in line with those studies in which MP emerged to be associated with worst levels of psychopathology among patients in diverse clinical populations [14,20]. However, our study is the first that supports empirically, using a cluster analysis, the specific discriminating role of MP as a specifier of different severity of symptoms. This confirms its clinimetric role as a specifier of “clinically significant distress” associated with symptoms of a psychiatric disorder, as Fava and colleagues recently suggested [17].

Consistently, when looking at correlation analysis, a significant relationship was found between observer-assessed MP and ED-core symptomatology and depressive symptoms. In terms of ED-related symptomatology in particular, greater disinhibited eating aspects, such as bulimic behaviors, have been found to be related to greater levels of MP when observer rated. The relationship between bulimic behaviors and higher emotion regulation difficulties is well known in the ED literature [63,64], making findings of the present study coherent with previous results finding an association between MP and emotional dysregulation and suppression [13,60]. Bulimic behaviors, such as binging and purging, have been found to be associated with worst quality of life, increased psychological distress, negative physical and psychological consequences, medical burden [65,66,67,68], and increased risk of suicide in EDs [69,70,71,72]. However, no significant difference in ED-core symptomatology was found when comparing ED patients with comorbid MP to those without MP. This result might be also due to an insufficient sample size or to other methodological issues (e.g., the high number of comparisons lowering statistical power).

Considering the specific evaluation of depressive symptoms, several associations emerged in ED patients between MP and a wide set of symptoms related to the depressive symptomatology. The joint use in our study of a comprehensive clinical interview for depression (CID-20) [38] with the DSM-5 diagnostic criteria for depressive disorders yielded important clinical insights into depressive disturbances associated with MP in our ED sample. Higher levels of MP showed a significant association not only with higher levels of severity of depression but also with greater suicidal tendencies. Depression in EDs is not only associated with the severity of eating symptomatology [73], but it is one of the major risks associated with suicidal ideation and attempts [74,75]. In this clinical population, suicidality may represent an escape from emotional pain [76] and is furthermore strongly related with interoceptive awareness deficits, making the individuals “out of touch” with their body [77] (p. 53) and helping them shift the attention from psychological pain to physical pain [33]. Although this association was not confirmed when comparing levels of suicidal ideation between patients with comorbid MP and patients without comorbid MP, the relationship between increased levels of MP and increased risk of suicide has been supported by the literature, and such correlation has been found independently of the severity of depressive condition, but at the same time, MP alone did not significantly predict suicide lethality [34]. For this reason, including the assessment of MP along with the evaluation of mood symptomatology is particularly warranted in this clinical population.

When comparing ED patients with MP to ED patients without MP, some significant differences emerged. ED individuals with MP more frequently reported a diagnosis of AN. This finding may be due, in part, to the majority of AN cases in our sample, but it might also be related to the greater severity characterizing AN compared to other ED diagnoses. Indeed, serious medical and psychological symptoms present in AN frequently persist even after recovery [78,79], and their underweight state is often accompanied by depression symptoms, such as depressed mood, social withdrawal, and irritability [26].

ED patients who reported MP also presented a more frequent comorbid diagnosis of depression according to the DSM-5 [26] when compared to those without MP and higher depressed mood symptoms (CID-20), supporting the well-known association between MP, depression, and suicidality [10,12,13,34] and, similarly, the well-established association between eating symptomatology and depression [31,36,80,81,82].

Furthermore, ED patients with comorbid MP also reported greater somatic-related depressive symptoms using CID-20 and BDI-II, supporting the overlap between MP and somatic pain [9,83,84] and the tendency of ED patients to express painful emotions through their body, turning the body into a “battleground” for unexpressed and unwanted emotions [53] (p. 17). Moreover, ED patients with comorbid MP reported higher levels of BDI-II cognitive depressive symptoms, such as guilt, self-criticism, and sense of failure [85], coherently with previous studies, which have found an association between MP and cognitive symptoms of depression [2]. This is also in line with the conceptualization of MP as a psychological condition arising from self-awareness of inadequacy and failure [11,86]. Lastly, ED patients with comorbid MP reported greater levels of general anxiety and insomnia measured by CID-20. Such association may also be due, even when not directly tested in our study, to the high degree of comorbidity with anxiety disorders present in EDs in which negative thinking, such as worry, co-occurs [87] together with body anxiety, eating, and avoidance behaviors related to food, body, and interpersonal situations [88,89,90].

While the well-known association between MP and depression was supported by our findings, it is also of clinical interest to remark that a subgroup of ED patients reported it also in the absence of a comorbid mood disorder—even though less frequently—confirming that MP may be considered as a distinct and isolated psychological condition from depression [2,10]. Further studies should target such important clinical aspects.

### 4.1. Implications

The presence of MP should be considered and included when assessing and treating patients with EDs due to its potential role as a specifier for DSM-5 “clinically significant distress” associated with ED diagnoses, paving the way for a more in-depth assessment that goes beyond the mere presence or absence of diagnostic symptoms as they are recognized in the standard psychiatric classification (DSM-5) [26]. An assessment that conversely considers the centrality of subjective phenomena (such as MP) may therefore demarcate clinical differences between ED patients [91], leading to a more individualized treatment [36]. Considering our results altogether, including the evaluation of MP in the assessment of individuals with EDs might help clinicians to identify patients experiencing higher distress that goes beyond ED core-related symptomatology, yielding incremental data to better evaluate the complex interplay between EDs and depression and the tendency to somatize emotions that characterize ED psychopathology through the body [53]. In particular, the assessment of MP in the context of eating disorders may have its clinical utility in the assessment of depression symptoms, which is particularly complex in this group of patients due to the overlap between somatic and physical symptoms that the two conditions have in common [36,80,92] and furthermore in detecting patients at higher risk for suicidal behaviors. Due to the high rate of suicide in ED individuals and the comorbidity with other psychiatric conditions [26,93], such distinction is clinically warranted in order to initiate targeted interventions and prevent significant morbidity.

### 4.2. Limitations and Future Directions

The reported findings should be interpreted in light of the limitations of the present study, namely the small sample size, the use of a mixed-ED sample, and the failure to not include a specific assessment of common psychiatric comorbidities, such as anxiety and personality disorders. The assessment of physical conditions (e.g., migraine or thyroid-related problems) that might influence the presence and severity of MP in ED patients was not included in the study [20], and patients and controls were not matched for social status and education. Moreover, depression in EDs, besides being one of the most common psychiatric comorbidities, shows a number of overlapping symptoms and similar cognitive features with EDs (e.g., weight loss, over-eating, sleep disturbance, fatigue, irritability, perfectionism, harm avoidance, etc.) [36,80,94,95,96], which makes differential diagnosis difficult [36].

Future research including a larger sample size is needed in order to further explore MP in ED individuals. Exploring MP in non-mixed ED samples (e.g., AN- or BN-only samples) is also warranted in order to observe how MP might present differently across different ED diagnoses. Longitudinal designs could also be used to investigate the role of MP in treatment response.

## Figures and Tables

**Table 1:** Socio-demographic characteristics of ED patients and controls.

Variables	Total ED Sample (*n* = 71) M ± SD	Control Sample (*n* = 90) M ± SD	*p*	Outpatients (*n* = 37) M ± SD	Inpatients (*n* = 34) M ± SD	*p*
Age	28.16 ± 11.29	29.36 ± 12.30	0.52 °	27.57 ± 12.57	28.82 ± 9.81	0.64 °
Education (years)	14.19 ± 3.21	15.40 ± 3.37	0.02 °	14.14 ± 3.05	14.25 ± 3.45	0.89 °
Marital status (% single)	86.96%	80.68%	0.34 #	94.59%	78.13%	0.09 #
Occupation % employed or student	74.63%	70.00%	<0.01+	94.44%	51.61%	<0.01+
% unemployed	23.88%	4.44%		2.78%	48.39%	
BMI						
AN (*n, %*)	(40, 56.3%) 15.28 ± 1.80			(16, 43.2%) 15.56 ± 1.77	(24, 70.6%) 15.09 ± 1.84	0.27 #
BN (*n, %*)	(13, 18.3%) 21.33 ± 1.91			(7, 18.9%) 21.69 ± 2.00	(6, 17.6%) 20.9 ± 1.87	0.31 #
BED (*n, %*)	(10, 14.1%) 36.20 ± 9.34			(8, 21.6%) 34.77 ± 10.27	(2, 5.9%) 41.21 ± 0.17	0.1 #
OSFED (*n, %*)	(8, 11.3%) 21.80 ± 9.62			(6, 16.2%) 18.03 ± 4.11	(2, 5.9%) 33.11 ± 14.93	0.051 #
Illness duration (months)	107.70 ± 110.18			77.14 ± 102.30	141.03 ± 110.24	0.01 °
Antidepressants (SSRI) use (%)	29.85%			23.53%	36.36%	0.25 +
Diagnosis of depression (MDD or PDD) (%)	55.88%			19.72%	33.80%	0.01 +

Notes: ° *t*-test for independent samples. + Pearson chi-Square. # Fisher’s exact test.; Abbreviations: AN, anorexia nervosa; BN, bulimia nervosa; BED, binge eating disorder; M, mean; MDD, Major Depressive Disorder; OSFED, other-specified eating or feeding disorder; PDD, Persistent Depressive Disorder; SD, standard deviation; SSRI, selective serotonin-reuptake inhibitors.

**Table 2:** Comparisons between ED patients and controls in mental pain using *t*-test for independent samples.

Measure (Range)	ED Patients (*n* = 71) M ± SD	Controls (*n* = 90) M ± SD	t(df)	*p*	Cohen’s d
MPQ	4.11 ± 2.61	1.68 ± 2.11	6.37 (133.15)	<0.01	1.04
Clinical assesment scale for mental pain	2.21 ± 1.37	0.56 ± 1.02	8.43 (124.18)	<0.01	1.39

Abbreviations: ED, eating disorders; M, mean; MPQ, Mental Pain Questionnaire; SD, standard deviation.

**Table 3:** Cluster clinical characteristics and differences calculated with *t*-tests.

Variables	Cluster 1 (*n* = 31) M ± SD	Cluster 2 (*n* = 31) M ± SD	t_(df)_	*p*
CASMP	1.23 ± 0.62	3.29 ± 1.16	5.191_(60)_	<0.0001
MPQ	2.52 ± 2.00	5.7 ± 2.24	5.220_(60)_	<0.0001
CID-20	39.19 ± 10.50	53.81 ± 11.64	5.990_(60)_	<0.0001
BDI-II	17.71 ± 11.36	31.42 ± 9.21	8.747_(60)_	<0.0001
EAT-40	41.35 ± 26.30	86.48 ± 46.50	4.704_(60)_	<0.0001

Abbreviations: BDI-II, Beck Depression Inventory-II; CASMP, Clinical Assessment for Mental Pain, CID-20, Clinical Interview for Depression; EAT-40, Eating Attitudes Test-40; MPQ, Mental Pain Questionnaire.

**Table 4:** Sociodemographic characteristics of ED patients with and without comorbid mental pain.

Variables	Mental Pain Presence (*n* = 31) M ± SD	Mental Pain Absence (*n* = 40) M ± SD	*p*
Age	28.70 ± 10.93	28.13 ± 11.58	0.83 °
Education (years)	14.12 ± 2.89	14.41 ± 3.32	0.72 °
Marital status (% single)	83.33%	89.47%	0.89 #
Occupation % employed or students	72.41%	62.16%	0.75 #
% unemployed	27.59%	24.32%	
BMI	18.27 ± 7.39	21.38 ± 9.08	0.13 °
Illness duration (months)	118.03 ± 115.38	102.16 ± 107.18	0.56 °
Antidepressants use (%)	27.59%	32.43%	0.67 +
ED diagnoses (%)			0.02 #
AN	32.9%	22.9%	
BN	7.1%	11.4%	
BED	1.4%	12.9%	
OSFED	2.9%	8.6%	
Outpatients (%)	17.14%	34.29%	0.058 +
Inpatients (%)	27.14%	21.43%	
Depressive disorder absence (%)	10.29%	33.82%	0.002 +
Depressive disorder presence (%)	33.82%	22.06%	

Notes: ° *t*-test for independent samples.+ Pearson chi-Square. # Fisher’s exact test.; Abbreviations: AN, Anorexia Nervosa; BED, Binge Eating Disorder; BMI, Body Mass Index; BN, Bulimia Nervosa, OSFED, Other Specified Feeding or Eating Disorders; M, Mean; SD, Standard Deviation.

## Data Availability

Due to the nature of this research, participants of this study did not agree for their data to be shared publicly, so supporting data are not available.

## Supplementary Materials

The following are available online at https://www.mdpi.com/article/10.3390/jcm10163584/s1, Table S1: Correlations between MPQ- mental pain, CID-20-mental pain item, EAT-40 and BDI-II and the CID-20-depression items in ED patients, Table S2: MANOVAs comparing ED patients with comorbid mental pain and ED patients without mental pain in EAT-40, BDI-II and CID-20 scores.
