# Supplementary material for: Mental Pain in Eating Disorders: An Exploratory Controlled Study
Source: J Clin Med. 2021 Aug 14;10(16):3584. doi: 10.3390/jcm10163584 (PMC8397208; doi:10.3390/jcm10163584)
Supplement: Supplementary file 1 [file jcm-10-03584-s001.zip › jcm-1327193-supplementary.pdf]

Table S1. Correlations between MPQ- mental pain, CID-20-mental pain item, EAT-40 and BDI-II and the CID-20-depression items in ED patients.

	Mental Pain Questionnaire	CASMP
**ED and Depressive Symptomatology Questionnaires**		
	r (p)	***r (p)***
**EAT- Oral Control**	.191 (.121)	.207 (.095)
**EAT-Bulimia and food preoccupations**	.234 (.057)	.329 (.007)
**EAT- Dieting**	.199 (.107)	.178 (.179)
**BDI-II Somatic Affect symptoms**	.593 (1.21)	.340 (.005)
**BDI-II Cognitive symptoms**	.587 (2.77)	.308 (.013)
**Clinical Interview for Depression Items**		
**CID-20-Depressed mood**	.341 (.004)	.400 (.001)
**CID-20-Guilt**	.302 (.012)	.081(.509)
**CID-20-Pessimism**	.255 (.034)	.108 (.375)
**CID-20-Suicidal tendencies**	.493 (<0.001)	.292 (.015)
**CID-20-Work and interests**	.262 (.030)	.290 (.016)
**CID-20-Energy and fatigue**	.186 (.125)	.256 (.034)
**CID-20-General anxiety**	.297 (.013)	.460 (<0.001)
**CID-20-Phobic anxiety**	.208 (.087)	.079 (.521)
**CID-20-Avoidance**	.255 (.035)	.064 (.601)
**CID-20-Somatic anxiety**	.332 (.005)	.407 (.001)
**CID-20-Irritability**	.130 (.286)	−.008 (.946)
**CID-20-Early insomnia**	.276 (.022)	.403 (.001)
**CID-20-Delayed insomnia**	.092 (.450)	.323 (.007)
**CID-20-Depressed appearance**	.281 (.019)	.092 (.452)
**CID-20-Environmental Reactivity**	−.145 (.234)	−.352 (.003)

Abbreviations: BDI-II, Beck Depression Inventory II; CASMP, Clinical assessment for mental pain; CID-20, Clinical Interview for Depression-20 items; EAT-40, Eating Attitude Test-40.

Table S2. MANOVAs comparing ED patients with comorbid mental pain and ED patients without mental pain in EAT-40, BDI-II and CID-20 scores.

	Multivariate Tests			
	**λ**	**df**	**F(p)**	η^2
**EAT-40 Scales**	.859	3.6	3.058 (**.036**)	.141
**BDI-II**	.851	2.00	4.88(**.011**)	.149
**CID-20 Scales**	.463	15.46	3.556 (**.004**)	.537
	Univariate Tests			

	Mental Pain Presence (N=31) Mean ± SD	Mental Pain Absence (N=40) Mean ± SD	F(p)	η^2
**EAT-Oral Control**	12.52 ± 7.57	9.21 ± 9.46	.906 (.345)	.015
**EAT -Bulimia and food preoccupations**	12.45 ± 6.46	8.03 ± 6.76	5.801 (.019)	.091
**EAT -Dieting**	25.76 ± 16.29	21.03 ± 16.60	.846 (.362)	.014
**BDI-II Somatic**	17.46 ± 6.07	11.79 ± 6.91	9.91 (**.003**)	.148
**BDI-II Cognitive**	11.93 ± 4.64	8.00 ± 6.16	6.21 (**.016**)	.098
**CID-20-Depressed mood**	4.31 ± 1.36	3.06 ± 1.45	10.713 (**.002**)	.151
**CID-20-Guilt**	3.59 ± 1.08	3.39 ± 1.27	.494 (.485)	.008
**CID-20-Pessimism**	3.10 ± 1.56	2.83 ± 1.64	.445 (.507)	.007
**CID-20-Suicidal tendencies**	2.69 ± 1.25	1.83 ± 1.23	7.335 (.009)	.109
**CID-20-Work and interests**	4.00 ± 1.60	2.61 ± 1.87	7.891 (.007)	.116

**CID-20-Energy and fatigue**	3.93 ± 1.58	3.06 ± 1.65	4.304 (.042)	.067
**CID-20-General anxiety**	4.21 ± 1.52	2.64 ± 1.37	17.630 (**.0001**)	.227
**CID-20-Phobic anxiety**	2.31 ± 1.71	2.36 ± 1.74	.073 (.788)	.001
**CID-20-Avoidance**	2.10 ± 1.80	2.39 ± 1.97	.401 (.529)	.007
**CID-20-Somatic anxiety**	3.55 ± 1.68	2.39 ± 1.46	9.080 (.004)	.131
**CID-20-Irritability**	2.34 ± 1.20	2.25 ± 1.36	.025 (.874)	.000
**CID-20-Early insomnia**	2.55 ± 1.68	1.56 ± 1.05	10.216 (**.002**)	.145
**CID-20-Delayed insomnia**	2.97 ± 1.61	2.03 ± 1.55	4.865 (.031)	.075
**CID-20-Depressed appearance**	2.21 ± 1.29	1.81 ± 1.14	1.130 (.292)	.018
**CID-20-Environmental Reactivity**	3.66 ± 1.63	4.61 ± 1.35	6.991 (.010)	.104

Notes: Comparisons adjusted for age, BMI, and illness duration; statistical significance in bold.
After Bonferroni corrections p values were set at 0.017 for EAT-40 subscales, 0.025 for BDI-II subscales, and 0.003 for CID-20 subscales.
Abbreviations: BDI-II, Beck Depression Inventory II; CID-20, Clinical Interview for Depression-20 items; EAT-40, Eating Attitude Test-40; M, mean; SD, standard deviation.
